# *Cryptococcus gattii* in an Immunocompetent Patient in the Southeastern United States

**DOI:** 10.1155/2016/8280915

**Published:** 2016-11-27

**Authors:** John W. Amburgy, Joseph H. Miller, Benjamin J. Ditty, Patrick Vande Lune, Shaaf Muhammad, Winfield S. Fisher

**Affiliations:** ^1^Department of Neurosurgery, University of Alabama at Birmingham, School of Medicine, Birmingham, AL, USA; ^2^School of Medicine, University of Alabama at Birmingham, Birmingham, AL, USA; ^3^Department of Veterans Affairs, Veterans Affairs Medical Center, Birmingham, AL, USA

## Abstract

Cryptococcal infections are seen throughout the United States in both immunocompromised and immunocompetent patients. The most common form is* C. neoformans*. In the Northwestern United States,* C. gattii* has received considerable attention secondary to increased virulence resulting in significant morbidity and mortality. There are no cases in the extant literature describing a patient with* C. gattii* requiring neurosurgical intervention in Alabama. A middle-aged immunocompetent male with no recent travel or identifiable exposure presented with meningitis secondary to* C. gattii*. The patient underwent 12 lumbar punctures and a ventriculoperitoneal shunt and required 83 days of inpatient therapy with 5-flucytosine and amphotericin B. The patient was found to have multiple intracranial lesions and a large intramedullary spinal cryptococcoma within his conus. Following an almost 3-month hospitalization the patient required treatment with oral voriconazole for one year. In the United States meningitis caused by* C. gattii* infection is not isolated to the Northwestern region.

## 1. Introduction

Cryptococcal disease in the United States has generally been attributed to* Cryptococcus neoformans*, which is most commonly seen in immunocompromised individuals. Cryptococcosis frequently presents as pneumonia but also has the capability to spread hematogenously to the central nervous system (CNS) causing meningitis and accumulations of yeast known as cryptococcomas. Cryptococcal disease is most often caused by two species complexes:* C. neoformans* species complex and the* C. gattii* species complex [1]. The taxonomy of* C. neoformans*/*C. gattii* species complex was recently updated in 2015. What were genotypes within these species complexes now have all been given a different species name.* Cryptococcus neoformans, Cryptococcus deneoformans*,* Cryptococcus gattii*,* Cryptococcus bacillisporus*,* Cryptococcus deuterogattii*,* Cryptococcus tetragattii*, and* Cryptococcus decagattii* are the new names [2]. The most common pathogen, previously known as* C. neoformans* var.* grubii,* is now known as* C. neoformans*. The serotype D isolates, previously named* C. neoformans* var.* neoformans*, are now members of the species* C. deneoformans*.

*C. neoformans* is the most common cryptococcal pathogen with a global distribution and predominantly infects immunosuppressed hosts. There is a greater predilection of* C. gattii* and* C. deuterogattii* to infect immunocompetent individuals than that of* C. neoformans* [1].* C. bacillisporus*,* Cryptococcus tetragattii*, and* Cryptococcus decagattii* are also nearly exclusively involved in infections among HIV-positive subjects. The ability of these species to spread is dependent on the yeasts' extracellular proteolytic activity, on melanin production, capsule formation, inositol, urease-activity, and ability to replicate inside macrophages. A deficiency in the protease produced by* C. gattii* as well as other properties limits its ability to cause cryptococcemia [3]. Instead,* C. gattii* forms larger inflammatory cryptococcomas that may require long-term antifungal therapy and neurosurgical interventions [1].

There are 5 known species of the* C. gattii* species complex (*Cryptococcus gattii, Cryptococcus bacillisporus, Cryptococcus deuterogattii, Cryptococcus tetragattii, and Cryptococcus decagattii*) [2]. As mentioned earlier,* C. gattii* and* C. deuterogattii* are frequently seen in immunocompetent patients but differ in their source:* C. gattii* is endemic to Australia and is a common source of infection in Aboriginal groups, while* C. deuterogattii* is the predominant form found in the recent Pacific Northwest outbreak [4, 5].* C. bacillisporus*,* C. tetragattii,* and* C. decagattii* are more frequently seen in immunocompromised patients [6]. Infections by* C. bacillisporus* occur more frequently and was found to be the major type in Californian HIV patients, while* C. tetragattii* is endemic to sub-Saharan Africa and mainly seen in HIV-infected individuals [5, 6].* C. decagattii* and* C. tetragattii* are unable to be distinguished by conventional PCR-fingerprinting and URA5-RFLP. Thus it is difficult to distinguish the two for specific distribution data [2].

Previously uncommon in North America, the* C. gattii* species complex disease emerged on Vancouver Island in 1999 and has since spread to the Pacific Northwest region of the United States [7, 8]. There have since been sporadic cases of* C. gattii* species complex cryptococcosis reported in the Southeastern United States, revealing the need to further define the populations at risk. For the first time, we report a case of* C. gattii* infection in an immunocompetent Alabama native with no recent travel or known exposure who developed severe meningoencephalitis requiring long-term antifungal therapy and neurosurgical intervention. The patient was previously in the military, however, and had extensive travel to Hawaii, Philippines, Thailand, Australia, Okinawa, and Hong Kong. His military experience was over a 5-year period ending in 1987. As this disease is associated with increase morbidity and mortality, this case highlights that the* C. gattii* species complex should be on the minds of those who treat cryptococcal disease in the United States.

## 2. Presentation of Case

A middle-aged male native to northern Alabama without a recent history of travel (but after further questioning revealed extensive travel to Hawaii, Philippines, Thailand, Australia, Okinawa, and Hong Kong over three decades earlier) was transferred to our hospital with the diagnosis of cryptococcal meningitis. The patient had a low complement levels (CH50) but was noted to have normal C5, MBL ELISA, C3, C4, and AH50. He was admitted with a history of cocaine use, but no injectable drug use. HIV testing including a viral load, gonorrhea, chlamydia, serum CMV antigen, urine histoantigens, and serum* Aspergillus* antigens were all negative. Four weeks prior to admission, the patient began to experience fevers, chills, headache, back pain, and vomiting. He was admitted to an outside hospital and a complete workup revealed cryptococcal meningitis via India ink stain of his cerebrospinal fluid (CSF). The patient was subsequently started on amphotericin B and 5-flucytosine for the cryptococcal meningitis, as well as vancomycin and ceftriaxone for possible concomitant bacterial meningitis.

**Figure 1:**
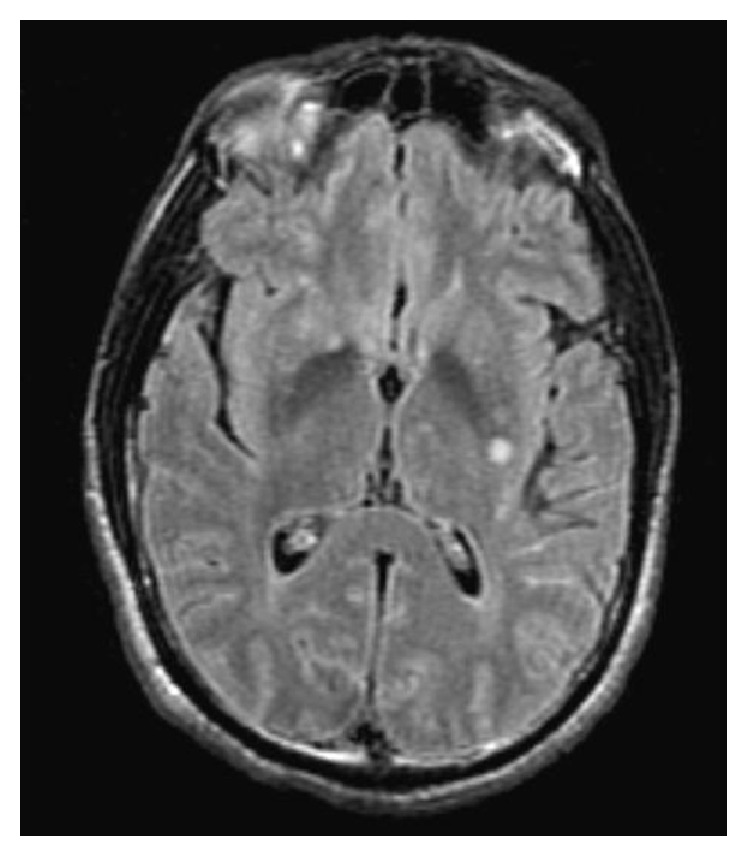
Revealing a cryptococcoma of the left basal ganglia and subcortical white matter.

The patient was transferred to our institution for treatment of cryptococcal meningitis, ultimately requiring 83 days of IV amphotericin B (Abelcet) and oral flucytosine therapy. Initial imaging revealed a peripheral right upper lobe lung nodule on chest CT, suggestive of cryptococcoma with negative acid-fast stain on bronchial lavage. MRI of the brain shown in Figure 1 was consistent for leptomeningitis and revealed a cryptococcoma of the basal ganglia and subcortical white matter.

Workup (+India ink stain, + cryptococcal antigen 1 : 512, and molecular typing of* Cryptococcus gattii* by CDC) determined the patient was infected with a member of the* C. gattii* species complex and further analysis was performed. The patient had a ventriculoperitoneal shunt placed for intractable headaches and elevated pressures on serial lumbar punctures.

**Figure 2:**
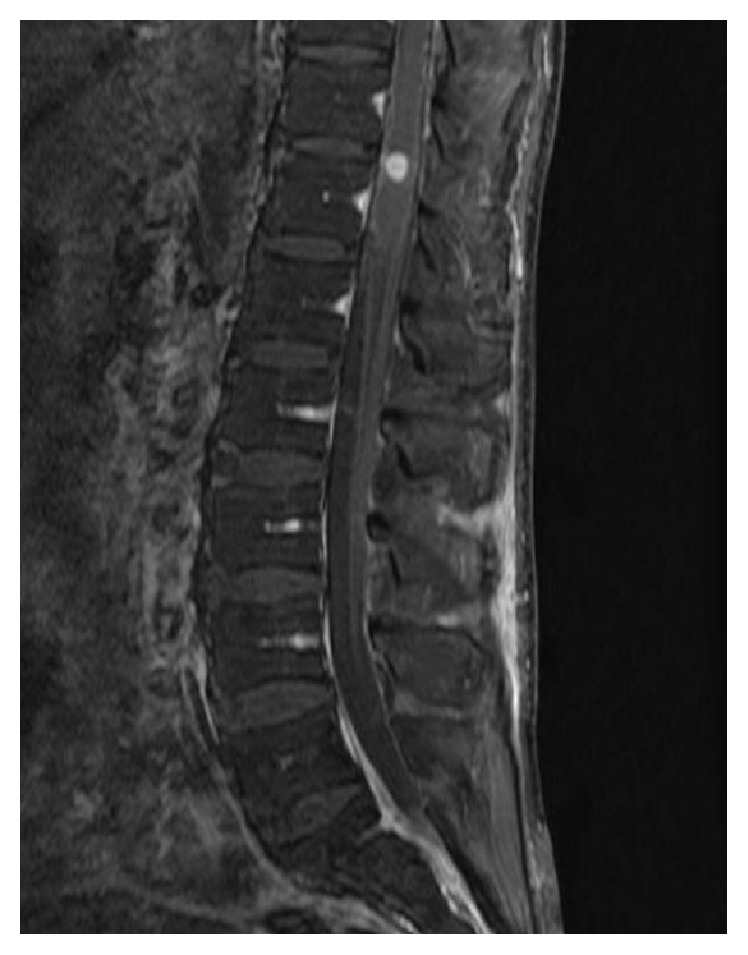
Reveals a T11-12 contrast-enhancing lesion consistent with a cryptococcoma.

Interferon-gamma therapy was initiated on day twenty for refractory infection and was administered three times per week. After 7 weeks of hospitalization, repeat imaging suggested worsening of intracranial cryptococcal disease, prompting an increase in amphotericin B from 5 to 6 mg/kg/day. During the following week, the patient complained of back pain radiating down his lower extremities that was exacerbated by amphotericin infusions. MRI of the spine shown in Figure 2 revealed a lesion at T11-12 consistent with a cryptococcoma.

It was decided to initiate corticosteroid therapy to treat cord edema as the patient was experiencing constipation and urinary urgency. Dexamethasone 4 mg every 6 hours was initiated, as it was believed that the patient had paradoxical antifungal-induced immune reconstitution inflammatory syndrome in setting of* C. gattii* meningitis.

After ten days of dexamethasone therapy, repeat MRI of the brain and spine showed improving white matter lesions with no evident spinal lesion, although meningeal enhancement was still present. Concurrent analysis of the CSF showed increased glucose and decreased protein, suggestive of an appropriate response to therapy. Final CSF cultures demonstrated no growth but India ink stain remained positive. After 83 days of therapy, amphotericin and flucytosine were discontinued and the patient was started on voriconazole 300 mg bid. The patient was monitored for three days and was subsequently discharged on the same voriconazole regiment with a dexamethasone taper.

## 3. Discussion

**Figure 3:**
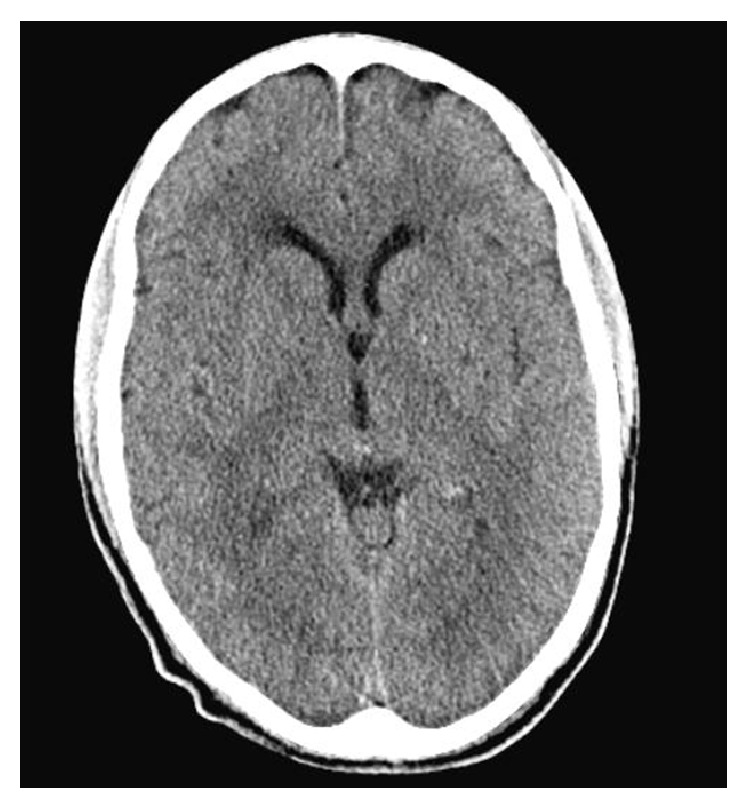
Reveals normal ventricular size on a CT head w/o contrast.

*C. neoformans* species complex and* C. gattii* species complex share some common virulent attributes but are also distinct in their pathogenicity, ecology, and epidemiology.* C. neoformans* has a global distribution and certain members of the* C. neoformans*/*C. gattii* species complex commonly infect predominantly immunocompromised individuals. In contrast, other members of the* C. gattii* species complex are more geographically restricted and commonly infect immunocompetent patients. Following inhalation of aerosolized yeast, these pathogens usually cause pulmonary disease with the capability of hematogenous dissemination to the CNS resulting in meningitis, cryptococcomas, and neurologic deficits [9]. Cryptococcal neurotropism to the basal ganglia stems from the ability of the yeast to capture and convert phenolic compounds to melanin, inducing protection from cytotoxic oxidants released from inflammatory cells [10]. Accumulation of the yeasts in these areas cause severe inflammation, dilation of the Virchow-Robin spaces, and impaired CSF reabsorption. This results in symptomatic intracranial hypertension despite a relatively normal appearance of the ventricular size as revealed on the head CT shown in Figure 3.

## 4. Conclusion

Most cases of* C. gattii* species complex in the United States have been in the Pacific Northwest and usually produce pulmonary disease [6]. The number of cases of the* C. gattii* species complex in the Southeastern United States are few and are frequently associated with travel to endemic areas. No member of the* C. gattii* species complex is considered to be endemic to the Southeastern United States and has never been isolated from environmental sources in the area [11].* C. gattii* species complex-members can be dormant for decades until the host's immune system becomes suppressed, and this patient may have contracted the pathogen during his time overseas in the military. Here we have reported for the first time a case of* C. gattii* species complex in an immunocompetent Alabama native that had no recent travel in over three decades, which required long-term antifungal therapy and neurosurgical intervention.
